# Supplementary material for: Hypotaurine promotes glioma cell invasion by hypermethylating the Wnt5a promoter
Source: PLoS One. 2025 May 15;20(5):e0312055. doi: 10.1371/journal.pone.0312055 (PMC12080842; doi:10.1371/journal.pone.0312055)

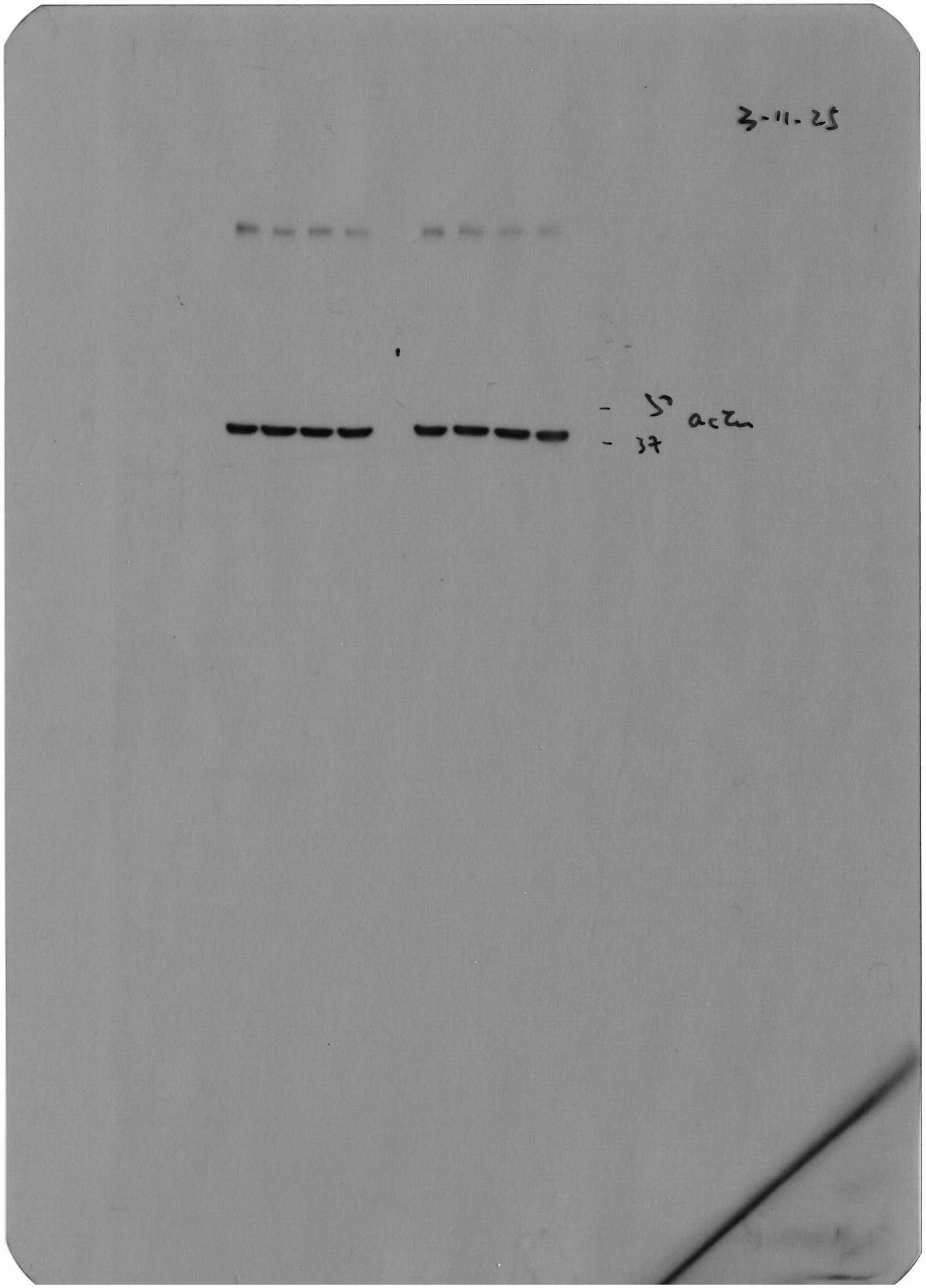
3-11-25
50
actin
37

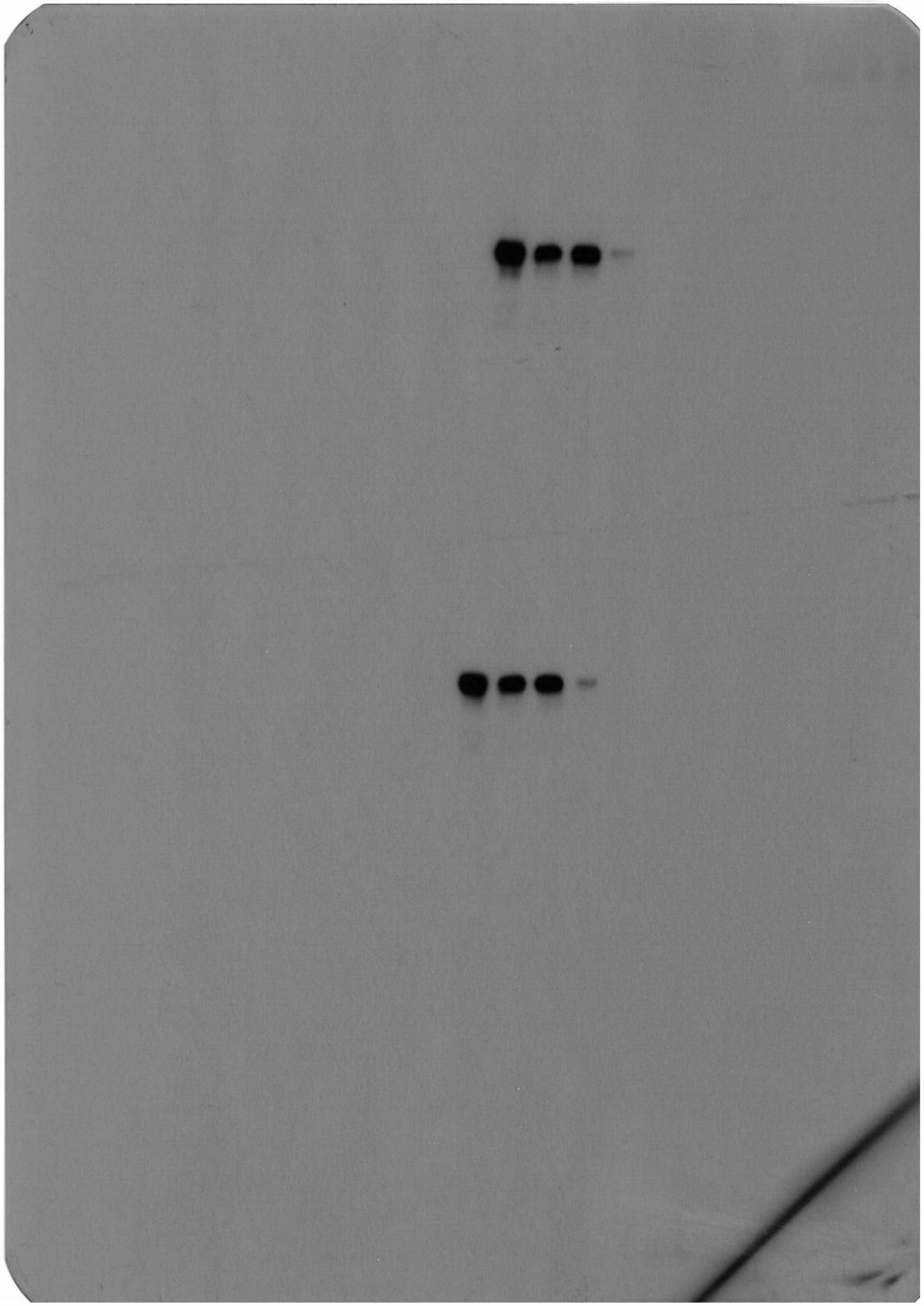

Supplement: S1 File — (PDF) [file pone.0312055.s001.pdf]
