# Supplementary material for: Hypotaurine promotes glioma cell invasion by hypermethylating the Wnt5a promoter
Source: PLoS One. 2025 May 15;20(5):e0312055. doi: 10.1371/journal.pone.0312055 (PMC12080842; doi:10.1371/journal.pone.0312055)
Supplement: Attachment — Submitted filename: renamed_79fa9.docx [file pone.0312055.s003.docx]

**Response to Reviewer #1 Comments**

**Reviewer Comment 1:** Clearly define the research objective in the opening paragraph of the introduction. State the primary question being addressed (the role of hypotaurine in glioma cell invasion) upfront to provide a clear framework for the reader.

**Response:** Thank you for this suggestion. We have revised the opening paragraph of the introduction to clearly define the research objective and state the primary question being addressed. The revision can be seen in Lines 41, 44-45, page 2 in the revised manuscript.

**Reviewer Comment 2:** Provide clear definitions and background information on key terms early in the paper. For example, explain the biological significance of hypotaurine and Wnt5a and their roles in cancer biology. This will make the paper accessible to a broader audience.

**Response:** We have added definitions and background information on hypotaurine and Wnt5a in the introduction. The revision can be seen in Lines 70-73, page 2 and page 82-87, page3 in the revised manuscript.

**Reviewer Comment 3:** Provide more mechanistic insights into how Wnt5a might function differently depending on cellular contexts. Include references to other studies where Wnt5a plays a role in cancer and provide a clearer explanation of its dual role as a tumor suppressor or promoter. This will clarify the ambiguity surrounding its function.

**Response:** We have expanded the discussion on Wnt5a’s dual role in cancer biology, citing relevant studies. The revision can be seen in Lines 254-270, page 9 in the revised manuscript.

**Reviewer Comment 4:** Expand the discussion on the potential clinical applications of these findings. Could targeting hypotaurine synthesis or Wnt5a methylation be a viable therapeutic strategy for glioma patients? Address the limitations and challenges in translating these findings from cell models to clinical practice.

**Response:** We have expanded the discussion on the clinical implications of our findings. The revision can be seen in Lines 312-321, page10 in the revised manuscript.

**Reviewer Comment 5:** Enhance the discussion by:

- Interpreting the results in light of existing literature on glioma invasion and epigenetic regulation. How do these findings fit into the broader context of glioma biology?
- Discussing the potential implications of these findings for therapeutic development (e.g., could hypotaurine inhibitors be used as a treatment strategy?).
- Offering possible explanations for the observed effects of hypotaurine on methylation and invasion, and suggesting follow-up experiments to further elucidate these mechanisms.

**Response:** We have enhanced the discussion by integrating the results with existing literature, interpreting the findings, and suggesting follow-up experiments. The revision can be seen in Lines 304-321, page 10 in the revised manuscript.

**Reviewer Comment 6:** Strengthen the conclusion by summarizing the key findings and emphasizing their potential impact on the understanding of glioma biology and the development of new therapeutic strategies. Additionally, clearly state any limitations of the current study and suggest areas for future research.

**Response:** We have revised the conclusion to summarize the key findings, highlight their significance, and address limitations and future directions. The revision can be seen in Lines 323-330, page 10 in the revised manuscript.

**Response to Reviewer #2 Comments**

**Reviewer Comment 1:** The authors highlight the tumor suppressor role of Wnt5a but they should discuss the dual role of Wnt5a in more detail.

**Response:** Thank you for pointing this out. We have expanded the discussion section to provide a more detailed explanation of Wnt5a's dual role in cancer biology. The revision can be seen in Lines 254-270, page 9 in the revised manuscript.

**Reviewer Comment 2:** The authors should discuss the specificity of hypotaurine's effects on demethylases.

**Response:** We appreciate this suggestion and have added a discussion on the specificity of hypotaurine's effects on demethylases. The revision can be seen in Lines 286-296, page 9 in the revised manuscript.

**Reviewer Comment 3:** A graphical abstract will help the readers.

**Response:** Thank you for this suggestion. We have created a graphical abstract summarizing the key findings of the study. The graphical abstract visually illustrates the role of hypotaurine in promoting glioma cell invasion and proliferation through its effects on Wnt5a promoter methylation and expression. The graphical abstract has been included as a separate figure in the manuscript.
